# Dietary vitamin E intake and risk of Parkinson's disease: a cross-sectional study

**DOI:** 10.3389/fnut.2023.1289238

**Published:** 2024-01-05

**Authors:** Xiaoqian Hao, Haiyan Li, Qinglian Li, Da Gao, Xiaoling Wang, Chunxiao Wu, Qizhang Wang, Meiling Zhu

**Affiliations:** ^1^Shenzhen Hospital of Integrated Traditional Chinese and Western Medicine, Shenzhen, Guangdong, China; ^2^The First Affiliated Hospital of Guangzhou University of Chinese Medicine, Guangzhou, Guangdong, China; ^3^The Fourth Clinical Medical College of Guangzhou University of Chinese Medicine, Shenzhen, Guangdong, China; ^4^Shenzhen Traditional Chinese Medicine Hospital, Shenzhen, Guangdong, China

**Keywords:** Parkinson's disease (PD), vitamin E intake, propensity score matching (PSM), the national health and nutritional examination surveys (NHANES), dietary

## Abstract

**Objective:**

Current evidence on the association between dietary vitamin E intake and the risk of Parkinson's disease (PD) is limited. The aim of the study was to explore the association of dietary vitamin E intake with PD in the United States among adults over 40 years.

**Methods:**

We conducted a cross-sectional study with data collected from National Health and Nutrition Examination Survey (NHANES) from 2009 to 2018. A total of the sample of 13,340 participants were included. To identify the different characteristics of the participants, we utilized propensity score matching (PSM) to reduce the effects of selection bias and confounding variables. Weighted univariate and multivariable logistic regression were used to examine the association between dietary vitamin E intake and PD before and after matching. Then, restricted cubic spline (RCS) was used to visually describe the possible non-linear relationships. Finally, we employed the subgroup analysis to further investigate the relationship between dietary vitamin E intake and PD.

**Results:**

According to the weighted univariate and multivariable logistic regression analysis, vitamin E intake was inversely associated with the risk of PD before and after matching. The results of RCS analysis revealed no non-linear inverse relationship between vitamin E intake and PD before and after matching. The subgroup analysis showed that age may influence the negative association between vitamin E and PD (*P* < 0.05 for interaction).

**Conclusion:**

Among participants over 40 years of age, vitamin E intake was negatively associated with the risk of PD. Our data may support the supplementation of vitamin E to be used as an intervention strategy for the occurrence of PD.

## Introduction

Parkinson's disease (PD) is a common progressive neurodegenerative disease, it is estimated that millions of people around the world has been affected by PD (1). Another study suggests that PD affects roughly 1% of people over 60 years (2). As the world population aging, PD prevalence will double in the next 2 decades (3). Many factors, including genes, environment and age, etc, play an important role in the pathogenesis and disease progression of PD (2). Studies have shown that neurological and nutritional factors may interact each other (4, 5). The main pathological characteristic of PD is the loss of dopaminergic neurons and the formation of Lewy bodies in substantia nigra, which involves abnormal aggregation of α-synuclein (α-syn), neuroinflammation, oxidative stress and mitochondrial dysfunction, etc (6, 7). When dopamine is released from synaptic vesicles into the cytosol or synaptic cleft, a large number of oxidants are produced in enzymatic and non-enzymatic reactions, so dopaminergic neurons are particularly susceptible to oxidative stress (8). Antioxidants in food were found to be neuroprotective, strengthening antioxidant pathways to counteracting neurodegenerative disease, such as PD (9, 10). However, the association between the progression of PD and the intake of vitamins remains unclear.

Vitamin E is an antioxidant and inhibits lipid peroxidation. Likewise, vitamin E is the only lipid-soluble chain-breaking antioxidant in human erythrocyte membranes and plasma (11, 12). Therefore, vitamin E may play an important role in the treatment of PD related to oxidative stress. Vitamin E is a group of compounds consisting of four tocotrienol (TT)- and four tocopherol (TP)-derivatives (13). TPs act as antioxidants by free radical scavenging and increasing the activity of antioxidant enzyme (14, 15). Certain TPs and TTs utilize various disturbances of the cellular and humoral immune systems to exert anti-inflammatory effects (16). Vitamin E compounds have indirect neuroprotective effects in PD due to neurotoxic potential of inflammation and oxidative stress. A clinical retrospective analysis shows that vitamin E levels in healthy participants are higher than those in PD patients (17). Despite numerous studies indicating that increased vitamin E intake is linked to a decreased risk of PD, a prospective clinical trial with PD patients found no evidence of vitamin E serving as an additional therapy (18, 19). There is controversy regarding the association between vitamin E intake and PD pathogenesis. Thus, we consider that the relationship between vitamin E and PD risk deserves further investigation.

Therefore, based on the National Health and Nutrition Examination Survey database (NHANES), we investigated the association between dietary vitamin E intake and PD by examining 13,340 participants from 2009 to 2018. The cross-sectional study can be used as a reference for the prevention of PD.

## Methods

### Database and survey populations

**Figure 1:**
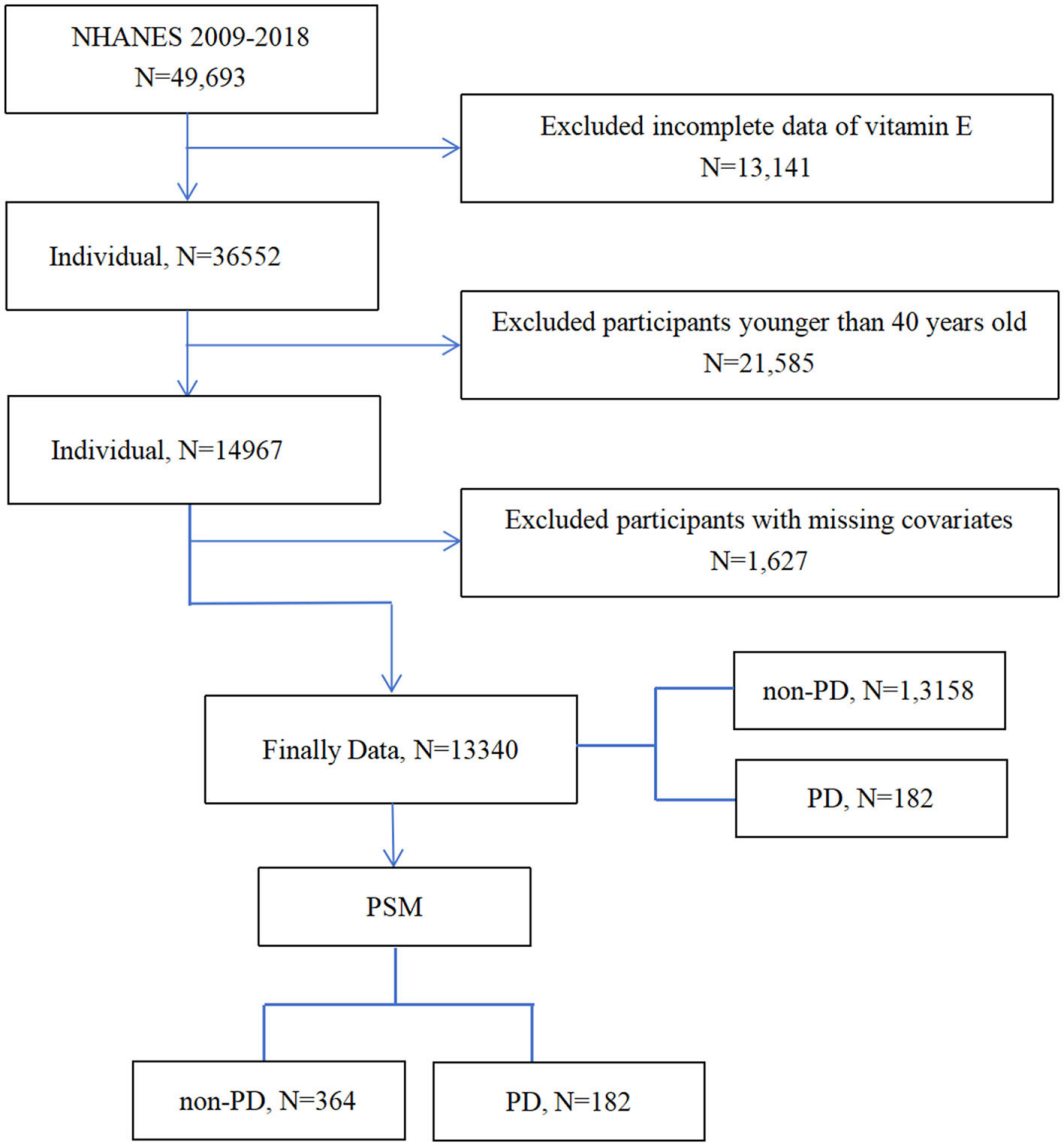
Flowchart of the participants' selection from NHANES 2009–2018.

In this study, data were come from the National Health and Nutrition Examination Survey website (https://www.cdc.gov/nchs/nhanes/index.htm). We collected publicly available NHANES's data from 2009 to 2018. A total of the sample of 13,340 participants were included in main analyses. Those who are under 40 years old were excluded. The integration process of entire data is shown in Figure 1.

### Study criteria of Parkinson's disease

In the NHANES database, we identified the participants as PD by using “Anti-Parkinson agents” (20, 21). This determination was made based on the responses to questions about prescription medication. This way of identification is affected by the medications and codes included in the NHANES that participants must be treated for PD to be identified with PD, and those who do not receive treatment were classified as not having PD.

### Dietary vitamin E intake measurement

Based on the data from the NHANES database, two recall interviews were conducted with the participants. Data on dietary vitamin E intake were collected through two 24-h recall interviews. The first interview was conducted at the NHANES mobile testing, while in the second recall interview, respondents were asked to complete 3–10 days of telephone interviews. To reduce errors in collecting dietary intake data, we took the average of the dietary intake data from these two interviews as the dietary vitamin E intake. It is worth noting that dietary vitamin E intake was only derived from food intake data and does not include the use of supplements.

### Assessment of covariates

In this study, demographic covariates included age, gender, race, marital status and education level. Chronic comorbidities included hypertension, diabetes and cardiovascular disease (CVD), which were confirmed by professional doctor diagnosis or a self-report questionnaire. Other potential factors included body mass index (BMI), energy intake, smoking status and alcohol status. BMI was calculated as weight (kg) divided by height squared (m^2^) by well-trained health technologists. The details of relevant definitions were shown in the Supplementary material.

### Statistical analysis

**Figure 2:**
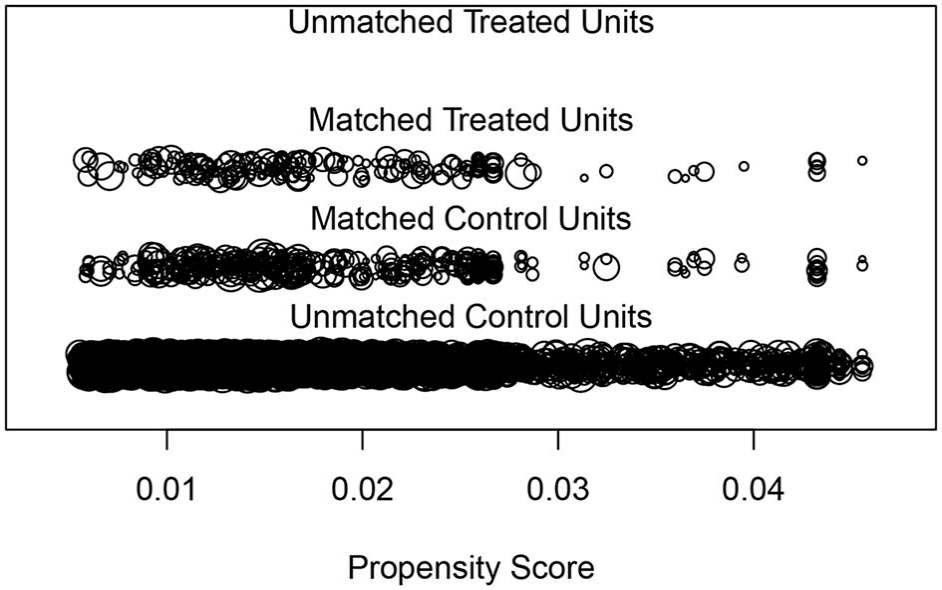
“Jitter” diagram of PSM.

To account for the complexities of the sampling survey, weighted analyses were conducted following the guidelines outlined by the NHANES. For categorical variables, frequency and percentages were reported, while for continuous variables, the mean and standard error (SE) were reported. We used chi-square tests to compare categorical variables, while we used a *T -* test to analyze the baseline characteristics of continuous variables. Propensity score matching (PSM) as a method can minimize the effects of possible biases and confounding variables in observational studies. In this study, confounding factors, including age, gender, race, hypertension and CVD, were used to matching. As shown in Figure 2, we used a 1:2 rule in the PSM method to balance cases and controls. Univariate and three multivariate logistic regression models were conducted to analyze the relationship between the dietary intake of vitamin E and the risk of PD before and after PSM. Restricted cubic spline (RCS) is one of the most common methods for analyzing non-linear relationships, and it is also recommended to be used as a tool to minimize residual confounding when adjusting continuous exposures (22, 23). We utilized RCS analyses based on multivariable logistic analysis in model 3 with four knots to visually describe the possible non-linear relationship between dietary vitamin E intake and PD risk, both before and after matching. We also conducted subgroup analyses based on age (40–50, 50–60, and ≥60 years), gender (male or female), race (Mexican American, non-Hispanic black, non-Hispanic white, other Hispanic, and other races), marital Status (non-single and single), education level (less than high school, high school, high school above), energy intake (tertile 1, T1; tertile 2, T2; tertile 3, T3), BMI (≤ 18.5, 18.5–25, 25–30 or ≥ 30 kg/m^2^), smoking status (never, former, and now), alcohol status (never, former, mild, moderate, and heavy), hypertension (no/yes), diabetes (no/yes), and CVD (no/yes) before and after PSM. Data extraction and analyses were conducted using EmpowerStats (version 3.4.3, www.empowerstats.com) and R (version 4.2.2, http://www.R-project.org).

## Result

### Characteristics of included participants before matching by PD

The study conducted statistical analysis on 13340 participants, included 182 patients with PD. The general characteristics of the participants are show in Table 1. The significant difference in age (*P* = 0.002), gender (*P* = 0.01), race (*P* < 0.0001), hypertension (*P* = 0.04) and CVD (*P* = 0.001) was observed between the PD group and non-PD group. The most important is that the significant difference in vitamin E intake mean (SE) between the PD and non-PD groups with 7.05 (0.38) and 8.85 (0.10), respectively (*P* < 0.0001).

**Table 1:** Baseline characteristics of participants from NHANES 2009–2018 before matching by PD.

**Variable**	**Total**	**NO-PD**	**PD**	***P-*value**
**Age**	58.06 (0.19)	58.00 (0.19)	61.99 (1.23)	0.002
**BMI**	29.65 (0.11)	29.65 (0.11)	29.40 (0.57)	0.67
**Energy (kcal)**	2029.59 (11.52)	2031.28 (11.61)	1914.29 (78.08)	0.14
**VE**	8.83 (0.10)	8.85 (0.10)	7.05 (0.38)	< 0.0001
**Gender**				0.01
Male	6,384 (47.86)	6,304 (46.96)	80 (34.49)	
Female	6,956 (52.14)	6,854 (53.04)	102 (65.51)	
**Race**				< 0.0001
Mexican American	1,744 (13.07)	1,731 (5.86)	13 (2.40)	
Non-Hispanic Black	2,956 (22.16)	2,933 (10.03)	23 (6.23)	
Non-Hispanic White	5,917 (44.36)	5,788 (73.50)	129 (87.80)	
Other Hispanic	1,357 (10.17)	1,345 (4.47)	12 (2.21)	
Other Race	1,366 (10.24)	1,361 (6.14)	5 (1.36)	
**Marital Status**				0.31
Non-single	8,352 (62.61)	8,245 (68.72)	107 (63.92)	
Single	4,988 (37.39)	4,913 (31.28)	75 (36.08)	
**Education**				0.69
< High school	1,346 (10.09)	1,329 (4.63)	17 (5.08)	
High school	4,745 (35.57)	4,678 (32.05)	67 (35.00)	
> High school	7,249 (54.34)	7,151 (63.32)	98 (59.92)	
**Smoke**				0.2
Never	7,040 (52.77)	6,948 (53.27)	92 (50.31)	
Former	3,973 (29.78)	3,919 (30.68)	54 (27.47)	
Now	2,327 (17.44)	2,291 (16.05)	36 (22.22)	
**Drinking**				0.45
Never	1,921 (14.4)	1,893 (10.48)	28 (12.79)	
Former	2,484 (18.62)	2,437 (14.98)	47 (20.18)	
Mild	5,152 (38.62)	5,081 (43.00)	71 (38.80)	
Moderate	1,887 (14.15)	1,868 (16.87)	19 (15.60)	
Heavy	1,896 (14.21)	1,879 (14.66)	17 (12.64)	
**Hypertension**				0.04
No	5,772 (43.27)	5,714 (49.19)	58 (39.08)	
Yes	7,568 (56.73)	7,444 (50.81)	124 (60.92)	
**Diabetes**				0.81
No	9,881 (74.07)	9,755 (79.78)	126 (80.44)	
Yes	3,459 (25.93)	3,403 (20.22)	56 (19.56)	
**CVD**				0.001
No	11,297 (84.69)	11,169 (87.50)	128 (77.94)	
Yes	2,043 (15.31)	1,989 (12.50)	54 (22.06)	

Data are presented as participant (percentage) for categorical variable and mean (SE) for continuous variables. P < 0.05 signify statistical significance.

### Characteristics of included participants after matching by PD

A comparison control group was established using closest-neighbor PSM (1:2) to analyze the relationship between the intake of vitamin E and the risk of PD. After PSM, data set included 364 participants in the control group and 182 participants in the PD group is show in Table 2. There were no significant differences in other covariates, while the significant difference between the PD and non-PD groups in dietary vitamin E intake mean (SE) with 7.05 (0.38) and 8.99(0.38) (*P* < 0.001) after matching.

**Table 2:** Baseline characteristics of participants from NHANES 2009–2018 after matching by PD.

**Variable**	**Total**	**NO-PD**	**PD**	***P*-value**
**Age**	62.56 (0.57)	62.83 (0.71)	61.99 (1.23)	0.58
**BMI**	30.01 (0.41)	30.29 (0.59)	29.40 (0.57)	0.33
**Energy (kcal)**	1941.74 (41.35)	1954.78 (53.66)	1914.29 (78.11)	0.69
**VE**	8.37 (0.30)	8.99 (0.38)	7.05 (0.38)	< 0.001
**Gender**				0.67
Male	239 (43.77)	159 (36.63)	80 (34.49)	
Female	307 (56.23)	205 (63.37)	102 (65.51)	
**Race**				0.96
Mexican American	40 (7.33)	27 (2.37)	13 (2.40)	
Non-Hispanic black	71 (13)	48 (5.92)	23 (6.23)	
Non-Hispanic white	384 (70.33)	255 (88.47)	129 (87.80)	
Other Hispanic	33 (6.04)	21 (1.62)	12 (2.21)	
Other race	18 (3.3)	13 (1.62)	5 (1.36)	
**Marital status**				0.67
Non-single	316 (57.88)	209 (66.45)	107 (63.92)	
Single	230 (42.12)	155 (33.55)	75 (36.08)	
**Education**				0.72
< High school	56 (10.26)	39 (6.14)	17 (5.08)	
High school	202 (37)	135 (31.23)	67 (35.00)	
> High school	288 (52.75)	190 (62.63)	98 (59.92)	
**Smoke**				0.12
Never	259 (47.44)	167 (46.94)	92 (50.31)	
Former	187 (34.25)	133 (37.89)	54 (27.47)	
Now	100 (18.32)	64 (15.17)	36 (22.22)	
**Drinking**				0.69
Never	77 (14.1)	49 (10.51)	28 (12.79)	
Former	127 (23.26)	80 (17.82)	47 (20.18)	
Mild	213 (39.01)	142 (41.37)	71 (38.80)	
Moderate	58 (10.62)	39 (13.26)	19 (15.60)	
Heavy	71 (13)	54(17.04)	17 (12.64)	
**Hypertension**				0.3
No	167 (30.59)	109 (33.38)	58 (39.08)	
Yes	379 (69.41)	255 (66.62)	124 (60.92)	
**Diabetes**				0.33
No	385 (70.51)	259 (76.76)	126 (80.44)	
Yes	161 (29.49)	105 (23.24)	56 (19.56)	
**CVD**				1
No	388 (71.06)	260 (77.95)	128 (77.94)	
Yes	158 (28.94)	104 (22.05)	54 (22.06)	

Data are presented as participant (percentage) for categorical variable and mean (SE) for continuous variables. P < 0.05 signify statistical significance.

### Association analysis of dietary vitamin E intake and PD before and after matching

#### Univariate logistics regression analysis of the association between dietary vitamin E intake and PD before and after matching

We use univariate logistics analysis to observe the association between the vitamin E, other covariates and PD. As shown in Table 3, age was positively correlated with PD occurrence and the effect value odds ratio (OR) and 95% confidence interval (CI) was 1.03 (1.01, 1.05) (*P* = 0.0014). Compared with male, female developing PD was a higher likelihood with an OR (95%) of 1.68 (1.17, 2.43) (*P* = 0.0068). People who had had hypertension, CVD had a higher risk of PD with a OR and 95% CI of 1.51 (1.03, 2.21) (*P* = 0.0368) and 1.98 (1.31, 2.99) (*P* = 0.0016), respectively. The most important is that dietary vitamin E intake was inversely associated with PD occurrence before and after matching. The OR (95% CI) of PD for dietary vitamin E intake was 0.91 (0.87, 0.96) (*P* = 0.0011).

**Table 3:** Univariate logistics regression analysis of the association between vitamin E intake and PD before and after matching.

**Characters**	**Unmatching**		**Matching**	
	**OR (95%CI)**	***P*** **-value**	**OR (95%CI)**	***P*** **-value**
**Age**	1.03 (1.01, 1.05)	0.0014	0.99 (0.97,1.02)	0.5808
**Gender**				
Male	Ref	Ref	Ref	Ref
Female	1.68 (1.17, 2.43)	0.0068	1.10 (0.71, 1.69)	0.6657
**Race**				
Mexican American	Ref	Ref	Ref	Ref
Non-Hispanic White	1.52 (0.74, 3.12)	0.2602	1.04 (0.41, 2.63)	0.9325
Non-Hispanic Black	2.92 (1.54, 5.51)	0.0015	0.98 (0.42, 2.30)	0.9624
Other Hispanic	1.21 (0.45, 3.20)	0.7073	1.35 (0.42, 4.31)	0.6106
Other Race	0.54 (0.17, 1.69)	0.2951	0.83 (0.19, 3.68)	0.803
**Marital Status**				
Non-single	Ref	Ref	Ref	Ref
Single	1.24 (0.82, 1.87)	0.3079	1.12 (0.67, 1.88)	0.669
**Education**				
< High school	Ref	Ref	Ref	Ref
High school	0.99 (0.51, 1.92)	0.9875	1.35 (0.54, 3.38)	0.512
> High school	0.86 (0.47, 1.58)	0.6331	1.16 (0.52, 2.59)	0.7215
**Smoke**				
Never	Ref	Ref	Ref	Ref
Former	0.95 (0.65, 1.39)	0.7857	0.68 (0.43, 1.06)	0.0852
Now	1.47 (0.89, 2.42)	0.1389	1.37 (0.69, 2.71)	0.3657
**Drinking**				
Never	Ref	Ref	Ref	Ref
Former	1.10 (0.64, 1.92)	0.7259	0.93 (0.45,1 0.93)	0.8448
Mild	0.74 (0.42, 1.29)	0.2930	0.77 (0.38, 1.57)	0.4695
Moderate	0.76 (0.35, 1.63)	0.4819	0.97 (0.40, 2.32)	0.9393
Heavy	0.71 (0.33, 1.50)	0.3674	0.61 (0.22, 1.69)	0.3361
**BMI**	0.99 (0.97, 1.02)	0.6780	0.98 (0.95, 1.02)	0.3254
**Hypertension**				
No	Ref	Ref	Ref	Ref
Yes	1.51 (1.03, 2.21)	0.0368	0.78 (0.49, 1.25)	0.296
**Diabetes**				
No	Ref	Ref	Ref	Ref
Yes	0.96 (0.68, 1.34)	0.8104	0.80 (0.51,1.25)	0.3291
**CVD**				
No	Ref	Ref	Ref	Ref
Yes	1.98 (1.31, 2.99)	0.0016	1.00 (0.62, 1.61)	0.9977
**Energy Kcal**	1.00 (1.00, 1.00)	0.1710	1.00 (1.00, 1.00)	0.6941
**VE**	0.91 (0.87, 0.96)	0.0011	0.91 (0.85, 0.97)	0.0042

#### Multivariable logistics regression analysis of the relationship between vitamin E intake and PD before and after matching

As shown in Table 4, three logistic regression models were used to investigate the connection between vitamin E intake and PD. Two adjusted models included: model 1 was adjusted for age, sex, race while model 2 was adjusted for age, sex, race marital status, education level, smoke, drinking, BMI, hypertension, diabetes, CVD and energy intake. There are all models displayed significant disparities before and after matching. Our results imply that vitamin E intake is closely connected with the occurrence of PD with OR and 95% CI of 0.91 (0.87, 0.96), 0.92 (0.87, 0.97), and 0.89 (0.84, 0.95) (*P* < 0.05) before matching. The significant association between vitamin E intake and PD occurrence was still observed after matching, which indicate that dietary vitamin E intake was inversely associated with the risk of PD and increasing dietary vitamin E intake could prevent PD.

**Table 4:** Multivariable logistics regression analysis of the association between vitamin E intake and PD before and after matching.

		**Unmatching**			**Matching**	
		**OR (95% CI)**	***P*** **-value**		**OR (95% CI)**	***P*** **-value**
Crude model	VE	0.91 (0.87, 0.96)	0.0011	VE	0.91 (0.85, 0.97)	0.0042
Model 1	VE tertile			VE tertile		
	T1	Ref	Ref	T1	Ref	Ref
	T2	0.76 (0.49, 1.19)	0.2418	T2	0.84 (0.47, 1.49)	0.5392
	T3	0.43 (0.28, 0.68)	0.0004	T3	0.44 (0.24, 0.80)	0.0086
	*P* for trend	0.0003		*P* for trend	0.0078	
		OR (95% CI)	*P*-value		OR (95% CI)	*P*-value
Model 2	VE	0.92 (0.87, 0.97)	0.0025	VE	0.91 (0.85, 0.97)	0.0040
	VE tertile			VE tertile		
	T1	Ref	Ref	T1	Ref	Ref
	T2	0.77 (0.49, 1.23)	0.2799	T2	0.82 (0.46, 1.47)	0.4969
	T3	0.46 (0.29, 0.72)	0.0013	T3	0.42 (0.23, 0.78)	0.0065
	*P* for trend	0.0012		*P* for trend	0.0057	
		OR (95% CI)	*P*-value		OR (95% CI)	*P*-value
	VE	0.89 (0.84, 0.95)	0.0011	VE	0.88 (0.81, 0.96)	0.003
	VE tertile			VE tertile		
	T1	Ref	Ref	T1	Ref	Ref
	T2	0.76 (0.46, 1.25)	0.2839	T2	0.76 (0.39, 1.46)	0.4021
	T3	0.40 (0.22, 0.71)	0.0027	T3	0.33 (0.14, 0.79)	0.0135
	*P* for trend	0.0027		*P* for trend	0.0124	

Crude model: No adjustments made for confounding factors. Model 1: Adjustments made for age, sex, race. Model 2: Adjustments made for age, sex, race marital status, education level, smoke, drinking, BMI, Hypertension, Diabetes, CVD and energy intake.

#### Non-linear relationship between dietary vitamin E intake and the risk of PD

**Figure 3:**
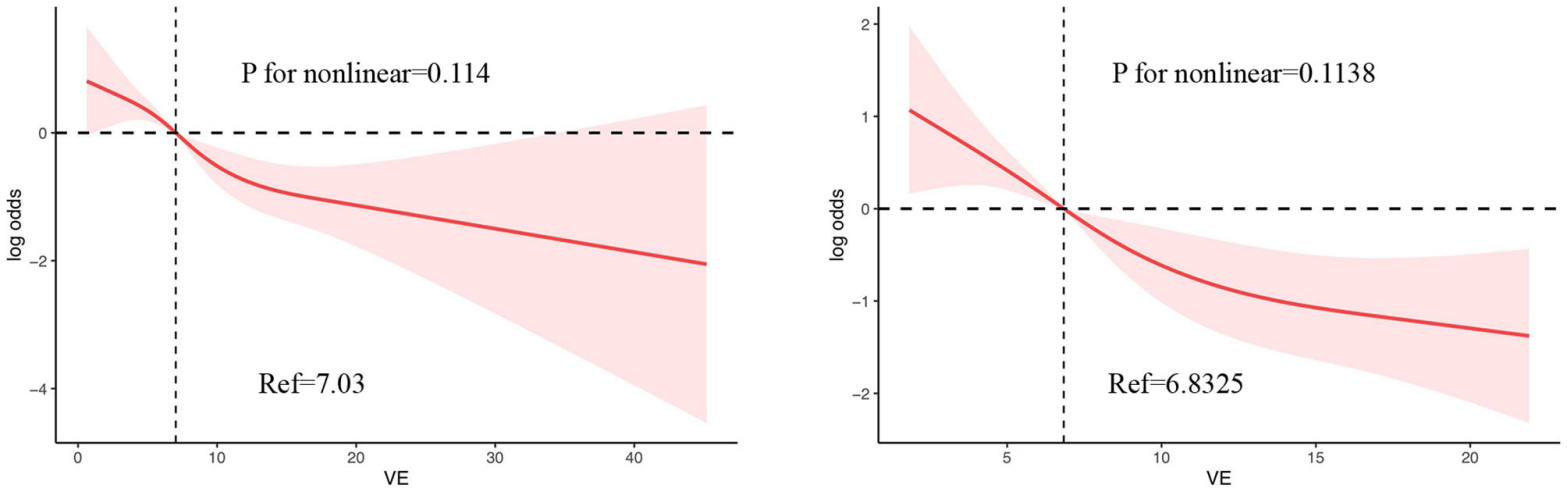
The RCS plot between vitamin E intake and PD before and after matching.

As evident in Figure 3, we used the restricted cubic spline RCS to visually describe the connection between vitamin E and PD before and after matching. The results of analysis revealed no non-linear inverse relationship between vitamin E intake and PD before and after matching. The OR of PD decreased when the dietary vitamin E intake increased.

#### Subgroup analysis before and after matching

**Figure 4:**
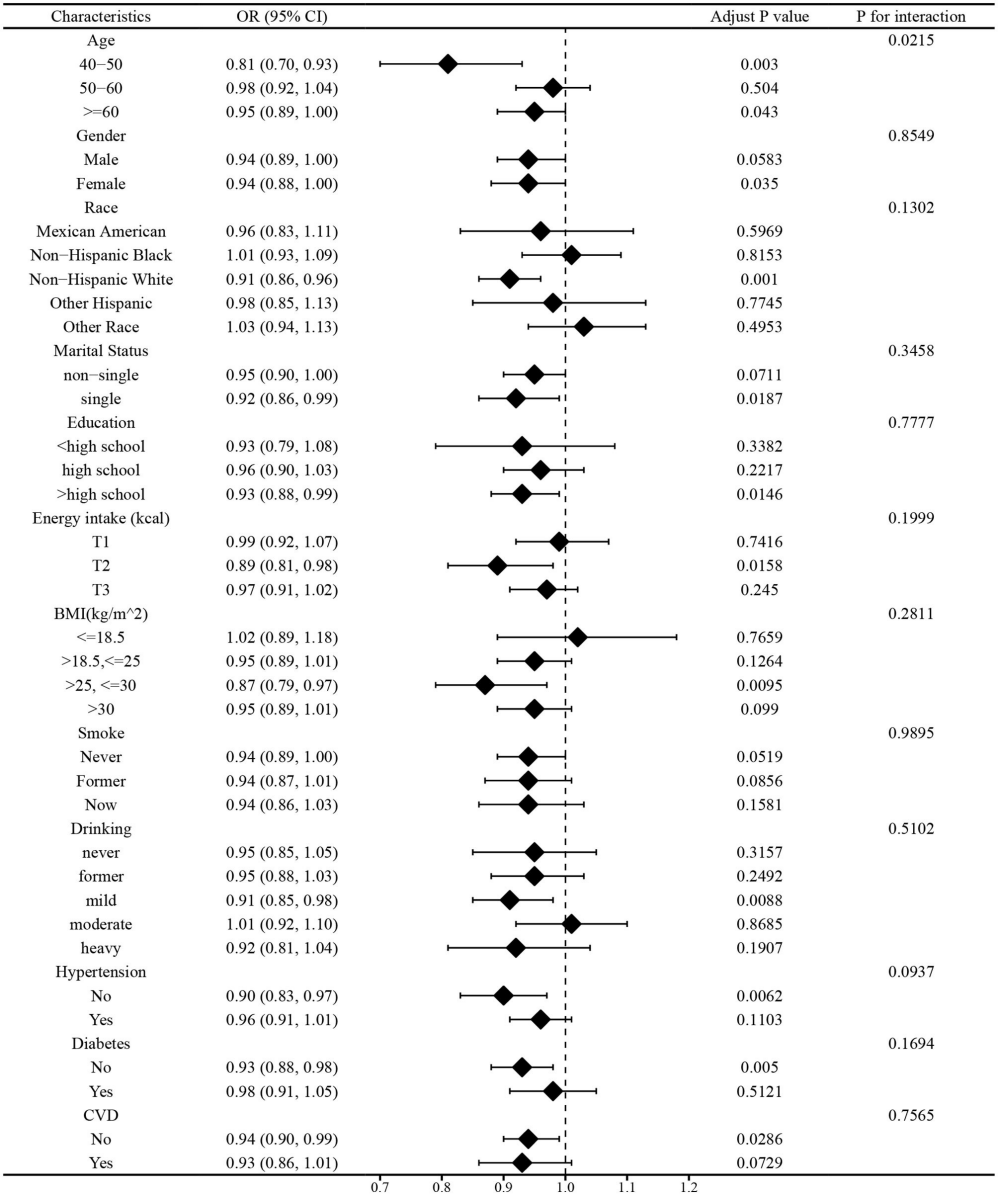
Subgroup analysis before matching.

**Figure 5:**
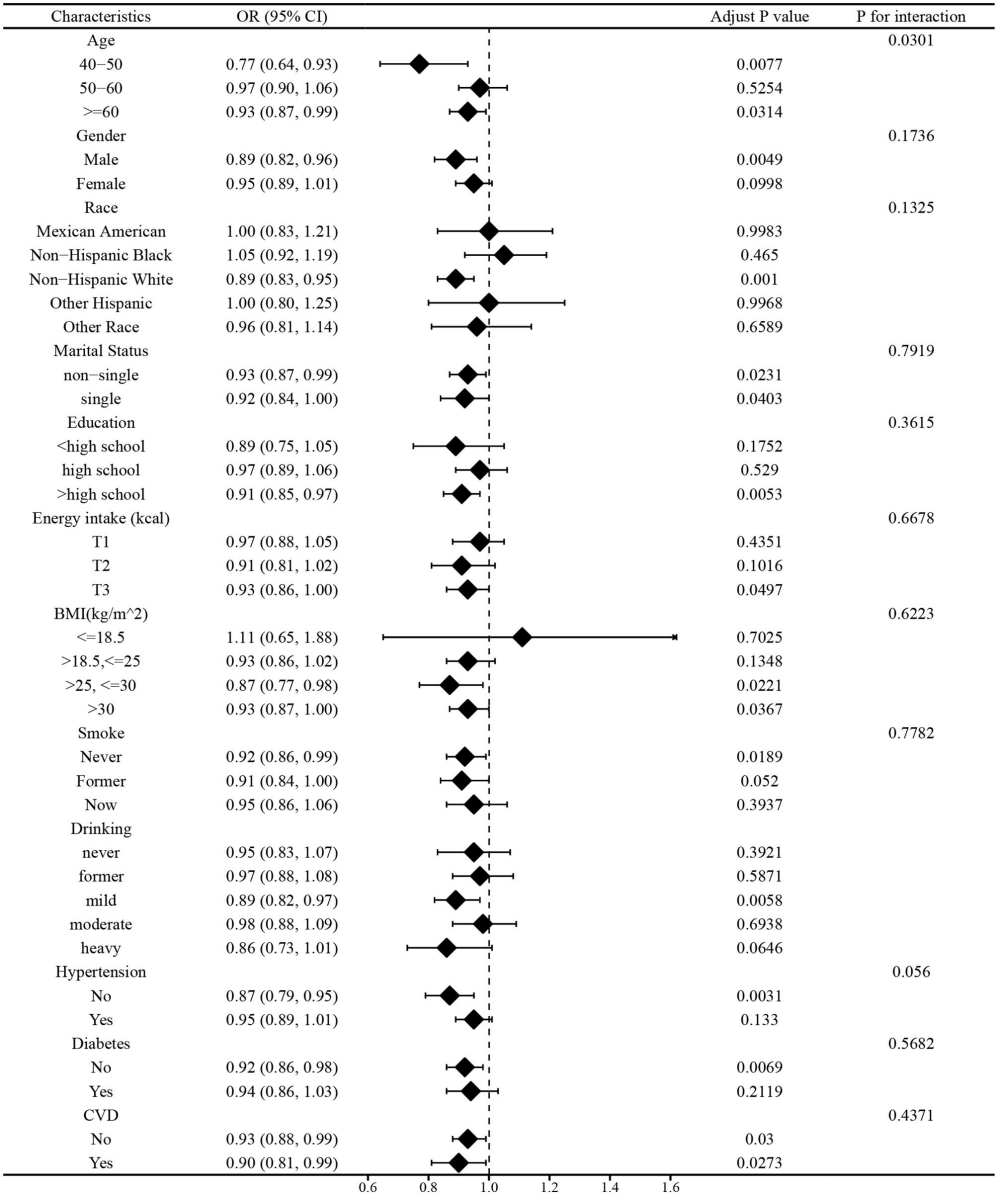
Subgroup analysis after matching.

In this study, we employed subgroup analysis to verify whether the relationship between vitamin E Intake and PD are affected by age, gender, race, marital status, education, energy intake, BMI, smoke, drinking, hypertension, diabetes, and CVD. As shown in Figures 4, 5, age could affect the negative association between dietary vitamin E intake and PD before and after PSM (*P* for interaction < 0.05). People aged 40–50 and over 60 had a strong relationship between vitamin E intake and PD before and after matching (*P* < 0.05). Generally speaking, the consistent results are considered to be reliable that before and after PSM matching. Therefore, the subgroup analysis shows that vitamin E intake was a significant protective factor in people who were aged 40–50 or over 60 years.

## Discussion

Vitamin E is an essential nutrient for the human bodily function (24). However, the association between vitamin E and PD remains unclear. It is necessary for us to investigate the relationship. According to the above, we conduct a cross-sectional study to examine the association between dietary vitamin E intake and PD. To reduce the effects of selection bias and confounding variables, we utilized PSM. The univariate and multivariate logistic regression showed that dietary vitamin E intake was inversely associated with the risk of PD. And RCS analysis revealed that the relationship between vitamin E intake and PD risk was no non-linear.

Vitamin E, as an antioxidant, can decrease the levels of oxygen-derived free radicals in neural tissue and regulate glutamate neurotoxicity to protect the brain (25). There are established diagnostic standards for the vitamin E deficiency of PD that are simple to administer. Patients with vitamin E deficiency will exhibit symptoms of cerebellar dysfunction and peripheral neuropathy, such as areflexia and limb ataxia (26). Experiments have demonstrated that these symptoms are significantly alleviated after vitamin E supplementation (27–29). Supplementing vitamin E can prevent detrimental effects of lipid peroxidation (LPO), eliminate free radicals generated by metabolism to safeguard cells. Likewise, adding vitamin E as a supplement can also improve motor deficits to enhance the quality of life for PD patients. Furthermore, a clinical retrospective analysis reveals that the levels of vitamin E are significantly higher in healthy individuals compared to age- and sex-matched 193 PD patients (17). A meta-analysis conducted in 2022 indicated a significant inverse association between increased vitamin E intake and the risk of developing PD, compared to individuals with lower vitamin E intake (30). In clinical trials, vitamin E therapy has shown potential in slowing down the progression of degenerative processes in patients with certain cortical diseases (31). However, the relationship between vitamin E and PD remains controversial, some studies suggest that long-term vitamin E supplementation can effectively prevent and treat PD (32), while others did not find any protective effect of vitamin E intake in PD patients (33, 34). Some studies found no significant difference in plasma vitamin E levels between PD patients and controls, suggesting that serum vitamin E concentrations were not associated with the risk of PD (35–37).

Reactive oxygen radicals can induce nigrostriatal lesions to construct a model of PD by contributing to the toxicity of 6-hydroxydopamine (6-OHDA) (8). Vitamin E has the potential to act as a substitute for the protective enzymes lacking in nigrka and locus coeruleus (LC) neurons. Furthermore, pretreatment with tocopherol has been shown to mitigate the toxic effects of 6-OHDA on the LC (38). Comparing to the rotenone induced model of PD, the increased number of tyrosine hydroxylase (TH) - positive neurons observed after pretreatment with a high dose of vitamin E can be attributed to its neuroprotective effect (39). Likewise, certain data analyses suggest that the reduction in apomorphine induced rotational behavior in rats may be attributed to the preventive effect of the vitamin on 6-OHDA induced dopamine depletion (40). The immunohistochemical analyses of TH in the study have shown that the reduction in the number of LC cells was smaller in the vitamin E pretreated lesioned group compared to the lesioned group (28). Lipid peroxidation can result in membrane damage, and vitamin E, being lipid soluble, acts as a chain-breaking antioxidant in biological membranes. By capturing the free radicals, vitamin E protects biological membranes from potential damage caused by these reactive species (41). It averts cellular damage by binding to free radicals and neutralizing their unpaired electrons, which is then converted to α-tocopherylquinone (39). There have been several pharmacological studies that primarily investigate the association between tocopherol derivatives (Toco-D) and PD. These studies suggest that Toco-D can improve catalepsy and locomotion by increasing the concentration of dopamine in the substantia nigra. Additionally, Toco-D may reduce oxidative stress levels and enhance neurotransmission to alleviate symptoms. Furthermore, it is speculated that Toco-D can inhibit inflammation by decreasing NF-κB levels in the brain, which could be one of the reasons contributing to the alleviation of PD (27).

An analysis was performed to investigate the non-linear association between variables and results. According to RCS analysis, there seems to be an association between the consumption of vitamin E in one's diet and the development of PD. Specifically, as the intake of vitamin E increases, the OR of developing PD decreases. Within a specific range of variation, vitamin E intake may serve as a protective factor for PD. Additionally, subgroup analysis demonstrated that individuals who were aged 40–50 and over 60 years exhibited dietary vitamin E intake as a protective factor. As a result of our study, we suggest that people take in foods rich in vitamin E, like the Mediterranean diet to prevent and treat PD.

Our study offers several notable advantages and implications. It is worth noting that it based on NHANES, which enhances credibility and ensures strong representativeness. Secondly, by employing PSM, we effectively reduce selection bias and enhance the credibility of our findings, thereby strengthening causal arguments and eliminating confounding factors often present in observational studies. Thirdly, through the utilization of RCS analysis, we were able to demonstrate the absence of no non-linear associations between dietary vitamin E intake and PD. Additionally, we conducted subgroup analysis to further explore the relationship between vitamin E and PD across different population groups. However, this study still has some limitations. In the first place, the data base we used which defined PD by the use of anti-Parkinson's drugs rather than a clinical diagnosis by professionals. The inclusion of individuals with mild PD symptoms in the healthy subjects group, as they may not require medication, and the inclusion of individuals with other neurological disorders in the PD group due to their use of Parkinson's drugs, can introduce bias in the analysis results. Given the cross-sectional design of our study, it is noted that no causal inferences can be made. Additionally, the presence of unidentified confounders may still have an impact, limiting the interpretation of our findings. Therefore, further prospective clinical studies are necessary to elucidate the intricate relationship between dietary vitamin E intake and PD.

## Conclusion

In summary, the findings of this study indicate that a higher vitamin E intake is linked to a reduced risk of PD to some extent. Additionally, the relationship between dietary vitamin E intake and PD risk exhibit no non-linearity. Considering the growing prevalence of PD and the potential neuroprotective properties of vitamin E, this discovery holds significant implications for both public health and clinical practice. It suggests that ensuring sufficient dietary vitamin E levels could be crucial in lowering the risk of Parkinson's disease. However, more extensive research is necessary to unravel the underlying mechanisms and thoroughly investigate the potential advantages and drawbacks of dietary vitamin E supplementation.

## Data availability statement

The datasets presented in this study can be found in online repositories. This data can be found at: https://www.cdc.gov/nchs/nhanes/index.htm.

## Ethics statement

The study was conducted in adherence to the ethical principles outlined in the World Medical Association Declaration of Helsinki. Prior to the interview and examination, all participants provided informed consent.

## Author contributions

XH: Data curation, Investigation, Methodology, Software, Writing – original draft. HL: Writing – original draft. QL: Writing – review & editing. DG: Writing – review & editing. XW: Writing – review & editing. CW: Writing – review & editing. QW: Project administration, Writing – review & editing. MZ: Project administration, Writing – review & editing.
