# Supplementary material for: Dietary vitamin E intake and risk of Parkinson's disease: a cross-sectional study
Source: Front Nutr. 2024 Jan 5;10:1289238. doi: 10.3389/fnut.2023.1289238 (PMC10799344; doi:10.3389/fnut.2023.1289238)
Supplement: Supplementary file 1 [file Data_Sheet_1.docx]

**Supplementary methods**

**1 Alcohol use**

Diagnostic criteria for alcohol use were as follows: never (had <12 drinks in lifetime); former (had ≥12 drinks in 1 year and did not drink last year, or did not drink last year but drank ≥12 drinks in lifetime); mild = c(1,2), 1 is for female and 2 is for male; moderate = c(2,3), 2 is for female and 3 is for male; or binge >=2 & binge <5; heavy = c(3,4), 3 is for female and 4 is for male; or binge >=5.

**2 Smoking status**

There were three categories of smoking: never, former, and current:(1) Never: smoked <100 cigarettes in a lifetime; (2) former: smoked >100 cigarettes in a lifetime and not currently smoking; (3) Now: smoked >100 cigarettes in a lifetime and smoking some days or every day.

**3 Diabetes**

Diagnostic criteria for diabetes were based on:(1) Physician diagnosis of diabetes. (2) Glycated hemoglobin HbA1c (%) >6.5. (3) Fasting blood sugar (mmol/L) >7.0.4. (4) Random blood sugar (mmol/L) ≥11.1. (5) 2-hour OGTT blood sugar (mmol/L) ≥11.1. (6)Use of diabetes medications or insulin.

**4 Hypertension**

Hypertension diagnosis was based on: (1) physician diagnosis of high blood pressure, (2) use of antihypertensive medications, or (3) abnormal blood pressure readings ( Repeat at least 3 times ).
